# A systematic model of the LC-MS proteomics pipeline

**DOI:** 10.1186/1471-2164-13-S6-S2

**Published:** 2012-10-26

**Authors:** Youting Sun, Ulisses Braga-Neto, Edward R Dougherty

**Affiliations:** 1 Department of Electrical and Computer Engineering, Texas A&M University, College Station, TX, USA; 2 Current affiliation: Department of Pathology, University of Texas M.D. Anderson Cancer Center, Houston, TX, USA; 3 Computational Biology Division, Translational Genomics Research Institution, Phoenix, AZ, USA; 4 Department of Bioinformatics and Computational Biology, University of Texas M.D. Anderson Cancer Center, Houston, TX, USA

## Abstract

**Motivation:**

Mass spectrometry is a complex technique used for large-scale protein profiling with clinical and pharmaceutical applications. While individual components in the system have been studied extensively, little work has been done to integrate various modules and evaluate them from a systems point of view.

**Results:**

In this work, we investigate this problem by putting together the different modules in a typical proteomics work flow, in order to capture and analyze key factors that impact the number of identified peptides and quantified proteins, protein quantification error, differential expression results, and classification performance. The proposed proteomics pipeline model can be used to optimize the work flow as well as to pinpoint critical bottlenecks worth investing time and resources into for improving performance. Using the model-based approach proposed here, one can study systematically the critical problem of proteomic biomarker discovery, by means of simulation using ground-truthed synthetic MS data.

## Background

### Mass spectrometry-based proteomics

Mass spectrometry (MS) is widely used for large-scale protein profiling with applications in biomarker discovery [1], signaling pathway monitoring [2,3], drug development, and disease classification [4]. In clinical applications of mass spectrometry, the number of samples available is usually in the range of tens to a few hundred (small sample size). The samples are analyzed by an MS instrument and transformed into a series of mass spectra containing hundreds of thousands of intensity measurements with signal generated by thousands of proteins/peptides (large feature dimension). This small-sample, high-dimensionality problem requires the experiment and analysis to be carefully designed and validated in order to arrive at statistically meaningful results.

### Motivation

The MS analysis pipeline consists of many steps, including sample preparation, protein digestion, ionization, peptide detection, protein quantification, and so on. The pipeline can be viewed as a noisy channel, where each processing step introduces some loss or distortion to the underlying signal and the end results are affected by the combined effects of all upstream steps. While individual components of the MS pipeline have been studied at length, little work has been done to integrate the various modules, evaluate them in a systematic way, and focus on the impact of the various steps on the end results of differential analysis and sample classification. In real experiments, it is not easy to decouple the compound parameter effects and determine the marginal influence of various modules on the end results, due to variations and the complicated nature of the work flow. Moreover, owing to contaminants and unknown or incomplete ground-truth, it is hard to meaningfully evaluate and compare results across different experiments. However, by employing a model-based approach, we may better understand the characteristics of the MS data, the contributions of the individual modules, and the performance of the full pipeline.

A key goal of MS-based proteomics is to discover protein biomarkers, which can be used to improve diagnosis, guide targeted therapy, and monitor therapeutic response across a wide range of diseases [1]. But to date, the rate of discovery of successful biomarkers is still unsatisfactory. This is due to challenges in the candidate discovery and biomarker validation phases, such as the high dynamic range of proteins [5,6], the tandem MS under-sampling problem [6], peptide redundancy and signal interference in the mass-to-charge domain [7], and inaccurate quantification of proteins [8,9]. Through the proposed model-based approach and by means of simulation using ground-truthed synthetic data, the problem of biomarker discovery can be studied and evaluated.

### Results

**Figure 1:**
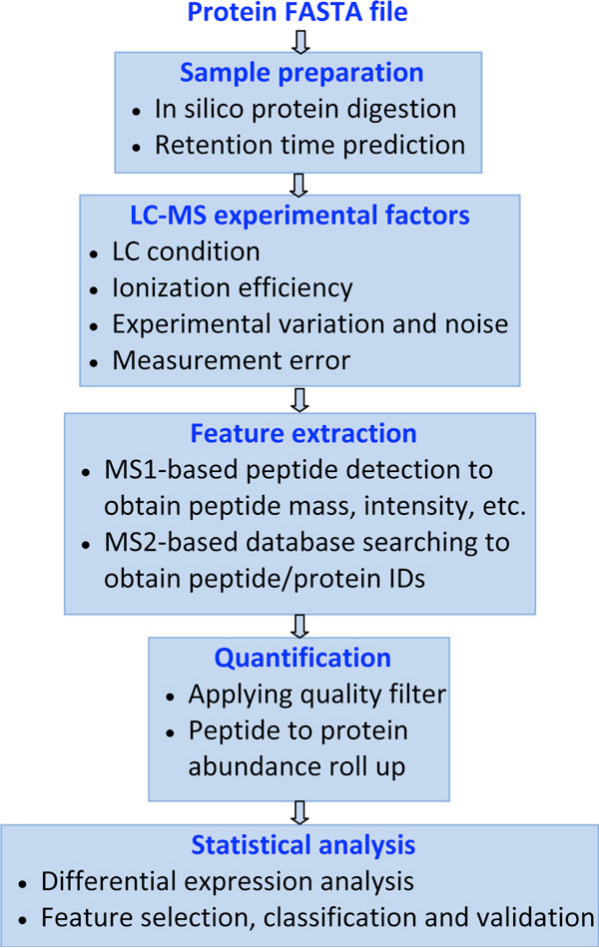
**The proposed MS-based proteomics workflow**. The proposed MS-based proteomics pipeline.

In this work, we propose to model the Liquid Chromatography (LC) coupled MS system by identifying critical factors that influence system performance. Different modules are identified and integrated into the framework (see Figure 1). The input of the pipeline can be any standard FASTA file containing proteins of interest. Here, we focus on analyzing protein drug targets downloaded from DrugBank [10], since LC-MS is an essential technology used to monitor these target proteins for drug development. We would like to point out that we are not trying to develop a detailed physical model for mass spectrometry as is, for instance, attempted in [11], which models the mass spectra generated by MALDI-TOF instruments. Rather, our purpose is to simulate the data flow realistically, but without descending into the physical parameters of the instrument itself. In addition, we do not focus only on MS data modeling, as done in [12], but we also address subsequent processes, including low level data analysis (e.g. peptide identification and quantification), and high level analysis (e.g. differential analysis and sample classification).

### Application of the proposed model

The proposed LC-MS proteomic pipeline model can be used to determine the working range of important parameters and may shed light on experimental design. Also, if knowledge of sample complexity, instrument configuration, system variation and detection accuracy is known beforehand, then by tuning corresponding parameters to their estimated values, the pipeline can be used to predict results on protein identification rates, protein differential analysis, quantification accuracies and classification performance. These results can be used to assess the efficacy of biomarker discovery in MS data.

## Methods

### Protein mixture model

In a typical label-free MS experiment, two sample classes (e.g. control vs. treatment) are considered. Assume each class has *M *samples and all samples share up to *N_pro _*possible protein species of a given proteome. Protein concentration in the pooled control sample is modeled by a Gamma distribution in accordance with the observations in [13]:

(1) $$\eta_{l}\sim \text{Gamma(}t\text{,}\theta ),\;l=1,2,...,N_{pro},$$

where *t *= 2 and *θ *= 1000 are the shape and scale parameters. The concentration has a dynamic range of approximately 4 orders of magnitude representing typical real-world scenarios. For the pooled treatment sample, expression levels of some proteins (e.g. biomarkers) may differ from those in the control sample, which can be captured by fold change:

(2) $$f_{l}=\left\{\begin{array}{cc} a_{l}, & \;\text{if protein }l\text{ is over-expressed}\\ \frac{1}{a_{l}}, & \;\text{if protein }l\text{ is under-expressed}\\ 1, & \;\text{otherwise} \end{array}\right)$$

where the fold change parameter, *a_l _*> 1, is sampled from a uniform distribution, as specified in the Results section.

Sample variation of each protein is modeled by a Gaussian distribution [14], with means *η_l _*and *η_l _f_l _*in the control and treatment sample classes, respectively. Considering the fact that protein expression levels are often correlated, the following multivariate Gaussian (MVG) distribution is appropriate to model the interactions among proteins and their concentrations. Let $c_{lj}^{pro}$ denote the molar concentration of protein species *l *in sample *j*, then we have

(3) $$c_{lj}^{pro}\sim \left\{\begin{array}{cc} \text{MVG}\left(\left[\eta_{1},\eta_{2},...,\eta_{N_{pro}}\right],\Sigma \right), & \;j\in \text{class 0}\\ \text{MVG}\left(\left[\eta_{1}f_{1},\eta_{2}f_{2},...,\eta_{N_{pro}}f_{N_{pro}}\right],\Sigma \right), & \;j\in \text{class 1} \end{array}\right)$$

where the covariance matrix Σ has a block-diagonal structure-proteins within the same block (e.g. proteins belonging to the same pathway) are correlated with correlation coefficient *ρ *and proteins of different blocks are uncorrelated [15]:

(4) $$\begin{array}{l} \sum ={\left[\sigma_{lj}^{2}\right]}_{N_{pro}\times N_{pro}},\\ \sigma_{lj}^{2}=\sigma_{ll}\sigma_{jj}r_{lj}, \end{array}$$

where *σ_ll _*is proportional to the control protein mean *η_l _*by a constant factor *ϕ_l _*(i.e., the coefficient of variation), and the correlation coefficient matrix is

$$R={\left[r_{lj}\right]}_{N_{pro}\times N_{pro}}=\left[\begin{array}{cccc} R_{\rho } & \;0 & \;\cdots & \;0\\ 0 & \;R_{\rho } & \;\cdots & \;0\\ \vdots & \;\vdots & \;\ddots & \;\vdots \\ 0 & \;0 & \;\cdots & \;R_{\rho } \end{array}\right],$$

where *R_ρ _*is a *D × D *matrix with 1 on the diagonal and *ρ *elsewhere. The correlation *ρ *and block size *D *are tunable parameters, with values specified in the Results section.

### Peptide mixture model

**Figure 2:**
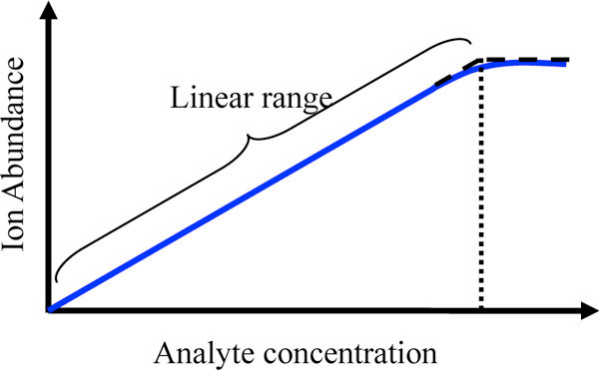
**The MS calibration curve**. MS ion signal as a function of analyte concentration in solution. The slope of the linear portion of the curve is the instrument response factor (i.e. instrument sensitivity). The curve departs from linear at high analyte concentration. A wider linear dynamic range is desired for quantitative analysis.

Before being analyzed by the MS instrument, proteins are usually digested into peptides. In the proposed simulation pipeline, *in-silico *tryptic digestion is performed, and retention time of peptide products is predicted using the PNNL Protein Digestion Simulator [16]. Different protein species may share the same peptide sequence. Thus, the molar concentration of peptide species *i *in sample *j*, $c_{ij}^{pep}$, is given by the following equation:

(5) $$c_{ij}^{pep}=\underset{k\in \Omega_{i}}{\sum }c_{kj}^{pro},\;i=1,2,...,N_{pep,}\;j=1,2,...,2M,$$

where the set Ω*_i _*comprises all proteins sharing the peptide species *i*, and *N_pep _*is the number of peptide species. The concentration $c_{ij}^{pep}$ is represented by ion abundance in MS data. Thus, the expected abundance readout *μ_ij _*of peptide species *i *in sample *j *can be modeled as

(6) $$\mu_{ij}=c_{ij}^{pep}e_{i}\kappa ,$$

where *e_i _*is a peptide efficiency factor similar to the one used in [17], and κ is the MS instrument response factor converting the original analyte concentration to the output ion current signal. The parameter *e_i _*is affected by many factors: first, various peptides differ in hydrophobicity, which mainly determines their efficiencies in passing through the liquid chromatography column. Then, upon entering the ionization chamber, peptides demonstrate great disparities in ionization efficiency, which is affected by sample complexity, peptide concentration and characteristics such as polarity of side chains, molecular bulkiness, and so on [18]. In addition, some amino acids at the N-terminal end of peptides have destabilizing effects that can reduce the efficiency factor. Although there are methods attempting to predict *e_i _*[17], they often neglect the fact that peptide efficiency and expected peptide ion abundance depend not only on the underlying peptide, but also on the combinational effects of other peptides present (e.g., LC elution competition, ion competition and suppression). In reality, it is unfeasible to predict *e_i _*for all possible peptide combinations. Thus, we model *e_i _*from a uniform distribution and evaluate a wide range of interval bounds in simulations -- we are not really interested in the precise value of *e_i_*, but rather we aim to examine how the dispersion of *e_i _*affects subsequent analysis. As for the parameter *κ*, it can be estimated through calibration and is related to the efficiency by which molecules are converted into gas-phase ions, the efficiency by which ions are transferred through various stages of the mass spectrometer, and how well experiment conditions are optimized. For a typical MS instrument, its response is linear for three to five orders of magnitude [18]. At high analyte concentration, instrument response plateaus because of detector saturation, restricted amount of excess charge, or limited space for ionization, as depicted in Figure 2. To account for instrument saturation, an upper limit, *sat*, is set for the expected abundance readout: *μ_ij _*= min(*μ_ij_*, *sat*).

### Peptide detection and identification

#### Peptide abundance

The actual abundance *v_ij _*of peptide species *i *in sample *j *is modeled as the expected abundance plus Gaussian noise:

(7) $$v_{ij}=\mu_{ij}+\varepsilon_{ij},$$

where

(8) $$\varepsilon_{ij}\sim \text{Gaussian(0,}\alpha \mu_{ij}^{2}+\beta \mu_{ij}),\;i=1,2,...,N_{pep},\;j=1,2,...,2M.$$

The sources of noise include variation in experimental conditions, instrument variance, thermal noise and measurement error. It is reported that the noise variance follows a quadratic dependence on the expected abundance [19], which is reflected by Eq. (8). The two parameters in the noise model, *α *and *β*, determine the noise severity. Their value can be estimated using replication analysis, as explained in [19].

In electrospray ionization, peptides can be multiply charged. But we do not model the charge distribution, considering the following facts: (1) Peptide charge distribution and the maximum charge states are complicated by many factors such as sample composition, analyte concentration and peptide conformation [20,21]. The distribution is hard to predict and has not been well characterized. (2) In order to get the abundance of a peptide, and further, its parent protein, the abundance of peptide charge variants will eventually be summed up. We omit the intermediate process since in reality many factors involved are not well understood.

#### Peptide detection

Peptide detection from mass spectra is not an easy task -- the observed peptide signals are corrupted by noise and may also be affected by signals of other peptides, and thus may deviate significantly from the expected pattern. The performance of a peptide detection algorithm on a specific MS instrument and the underlying signal-to-noise ratios (SNRs) ultimately affect the number of detected true positives, i.e., the true positive rate (TPR), as shown in [22-25]. The SNR is defined as the ratio of signal power to noise power, i.e., $SNR≜E{\left[v\right]}^{2}/\text{Var(}v\text{) = 1/(}\alpha \text{ + }\frac{\beta }{\mu })$, see Eqs. (7)-(8). It can be seen that SNR increases as signal strength *μ *increases. The relationship between TPR and SNR can be approximated by a polynomial function, for algorithms such as those in [22,23,25]:

(9) $$TPR=k\times SNR^{p}+b,$$

where *b *represents the worst TPR when the SNR approaches zero.

Besides *SNR*, signal interference and mass resolving power may also have considerable impact on TPR [12,23]. Over the years, much effort has been made towards enhancing instrument resolution, leading to improved mass accuracy, better separated MS peaks, and less convoluted peptide signals. But for complex samples, substantial overlapping of peptide signals is still frequently encountered, due to peptide isoforms or co-elution. It has been reported that if two peptides have overlapping signal regions, some detection algorithms may fail to report one of them even when the underlying SNRs are high, while other algorithms are shown to be superior in the detection of overlapping peptides [22]. To account for signal interference, we modify Eq. (9) by introducing an overlapping factor *o_ij_*, so that the TPR of peptide species *i *in sample *j *becomes

(10) $$TPR_{ij}=\left(k\times SNR_{ij}^{p}+b\right)\times o_{ij},\;o_{ij}\leq 1.$$

For algorithms such as NITPICK [24], BPDA [22] and BPDA2d [23], which are effective in detecting overlapping peptides, the overlapping factor *o_ij _*can be approximated by 1, whereas for algorithms that are ineffective in detecting convoluted peptides, *o_ij _*is assumed to be inversely proportional to the number of overlapping peptides, which is a function of the sample composition and the mass resolution. In our simulation, two peptide species *i*_1 _and *i*_2 _are said to overlap if their mass and retention time (RT) are close, in the sense that

(11) $$\frac{\left|mass_{2}-mass_{1}\right|}{mass_{1}}<\frac{1}{\text{mass resolution}}\;\text{and}\;\frac{\left|RT_{1}-RT_{2}\right|}{\#\text{scans}}<0.005.$$

#### Peptide identification

The output of the MS1-based peptide detection algorithm is a list of detected peptides annotated by monoisotopic mass, retention time, abundance, and so on. To obtain peptide sequence information, i.e. peptide identification, which can be used to infer the parent protein from which the peptide was digested, database searching is required. To do so, the acquired MS/MS (MS2) spectra are searched against a protein database containing theoretical MS2 spectra generated from *in-silico *digested peptide sequences by popular software such as SEQUEST [26] and Mascot [27].

Several machine learning methods have been proposed to predict the probability (i.e., identifiability) of a peptide being identified through MS2 database searching [14,28]. These methods try to extract the common trends residing in peptide identifiability that can be explained by peptide sequence-specific properties. Their successful application may suggest that the peptide sequence largely affects the chance of a peptide getting selected for MS2 analysis, whether the peptide can be sufficiently fragmented, and the quality of its fragmentation spectra. In our simulation, the identifiability *p_i _*of the true peptide species *i *is predicted by the APEX software [14], trained on the human serum proteome [29], and whether peptide species *i *in sample *j *is identified or not through database searching is determined by the outcome of a Bernoulli trial with success rate *p_i_*.

#### Linking of detection and identification results

For both MS1-based and MS2-based algorithms, sources of error exist that give rise to false positives (FPs). For the former, error sources include shot noise, abundance measurement error, signal interference, and so on. For the latter, co-eluting precursor ions, spectra matching ambiguity, or post-translational modifications may all lead to false identifications. By confronting the results of the two orthogonal algorithms (i.e., a feature is treated as a true positive if it is reported by both algorithms), dubious features reported by either algorithm can be filtered out.

### High-level analysis

#### Peptide to protein abundance roll-up

As demonstrated in the previous sections, each step of the MS analysis pipeline introduces a degree of loss or distortion to the underlying true signal. Thus, "decoding" protein abundance from observed peptide abundance corrupted by noise is nontrivial. To reduce noise, three levels of filtering are applied: (1) only unique peptides that exist only in one protein of the analyzed proteome are kept; (2) peptides with large missing value rates (larger than 0.7) are filtered out, since low reproducibility may be a red flag for false identifications; (3) among the remaining peptides, those having sufficiently high correlations (larger than 0.6) with other peptides digested from the same protein are retained. The estimated abundance of protein *l *in sample *j *is then obtained by averaging the abundances of its children peptides that pass the previous filters; if less than two peptides pass the filters, the estimated protein abundance is set to zero. The estimated protein concentration is calculated by dividing the estimated protein abundance by the instrument response factor *κ*.

Quantification accuracy can be assessed by the commonly adopted mean quantification error, defined by

$$qerr≜\frac{\overset{N_{pro}}{\underset{l=1}{\sum }}\overset{2M}{\underset{j=1}{\sum }}|c_{lj}^{prot}-{ĉ}_{lj}^{prot}|/c_{lj}^{prot}}{2M\;N_{pro}},$$

where $c_{lj}^{prot}$and ${ĉ}_{lj}^{prot}$are the original and estimated concentrations of protein *l *in sample *j*, respectively.

#### Differential expression analysis

Differential expression analysis is performed via a two-sample t-test with equal sample size and variance. The t statistic (or t score) is calculated as below:

$$t_{l}≜\frac{\left|m_{l}^{1}-m_{l}^{0}\right|}{\sqrt{\frac{Var_{l}^{1}+Var_{l}^{0}}{M}}},$$

where the superscripts identify the two classes, and *m_l _*and *Var_l _*represent the estimated class mean and variance of the abundance of protein *l*, respectively. The standard 0.05 significance level is used to detect differentially expressed markers.

#### Feature selection and classification

In the simulation, t-test feature selection is first performed to reduce the data dimension, by selecting the top 20 differentially expressed features. Then two classifiers, namely K-nearest neighbor (KNN, K = 3) and linear discriminant analysis (LDA) are trained using the observed protein expression data. Classification performance is validated by independent ground-truth (testing) data sets (each with 1000 samples, generated from the same data model), and the classification error is recorded. In addition, the KNN and LDA classification error on the original protein data (before entering the MS analysis pipeline) is obtained using a similar approach. The latter may serve as a benchmark to gauge how much loss in classification performance the analysis pipeline has introduced.

## Results

To illustrate the application of the proposed pipeline model, a FASTA file containing around 4000 drug targets (human proteins) was compiled from DrugBank [10], which serves as the underlying proteome to be studied. In each run, 500 background proteins along with 20 marker proteins are randomly selected from the proteome to serve as the input of the pipeline. For each experimental setting studied, the simulation is repeated 50 times. We are interested in the effects of various factors on quantification, differential analysis, and classification. The study should be carefully designed to minimize parameter confounding effects. Thus, while examining the effects of one parameter, we either fix the values of other parameters, or try to eliminate their effects. Parameter configurations are given in Table 1, unless otherwise mentioned.

**Table 1:** Proteomics pipeline model summary

Parameters	Default values
No. of classes	2
Sample size of each class	*M *= 50
Proteome	Homo sapiens
No. of marker proteins	20
No. of non-markers	500
Protein block size	*D *= 2
Protein block correlation	*ρ *= 0.6
Fold change	*a_l _*~ Unif(1.5, 2)
Instrument response	*κ *= 5
Instrument saturation effect	*sat *= Inf
Noise level	*α *= 0.03, *β *= 3.6
Peptide efficiency factor	*e_i _~ *Unif(0.1, 1)
Peptide detection algorithm	*b *= 0, *k *= .0016, *p *= 2
No. of MS2 replicates	1

### Sample characteristics

#### Effect of peptide efficiency factor

**Figure 3:**
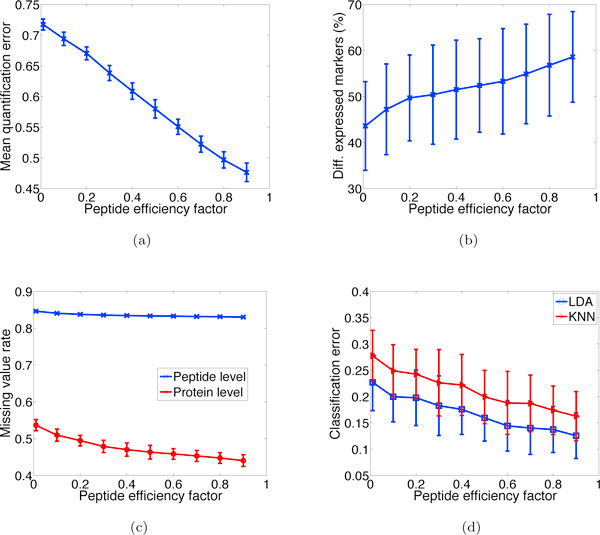
**Effect of peptide efficiency factor**. Various quantities plotted as a function of the lower bound of peptide efficiency factor (the upper bound is fixed at 1). (a) Mean quantification error as defined in Eq. (12). (b) Percentage of observed differentially expressed marker proteins at a 0.05 significance level. (c) Missing value rates at the protein and peptide levels. (d) Classification error rates given by LDA and KNN classifiers, respectively.

Though the exact distribution of the peptide efficiency factor *e_i _*is unknown, we evaluate a wide range of values and try to find the common trend. It can be seen from Figure 3(a) that as the lower bound of *e_i _*increases, the quantification error decreases. This is expected since more ions can be detected by the instrument and transmission loss is reduced as efficiency increases. Figure 3(b) suggests that the percentage of observed differentially expressed proteins is positively correlated with *e_i_*: this may be explained by the fact that as *e_i _*increases, fewer missing values occur at the peptide level, and more proteins can be quantified in more samples, as can be seen in Figure 3(c), resulting in more markers being detected by the differential expression test. Figure 3(d) shows that the additional detected markers help to improve classification accuracy by decreasing the classification error.

#### Effect of protein abundance

The distribution of in-solution protein abundance can affect various detection results [30]. While high-abundance proteins are easily detectable, low-abundance proteins are hard to detect since their signals are more likely to be buried in background noise. Hence, improving detection of low-abundance proteins has become a central issue in proteomic research.

**Figure 4:**
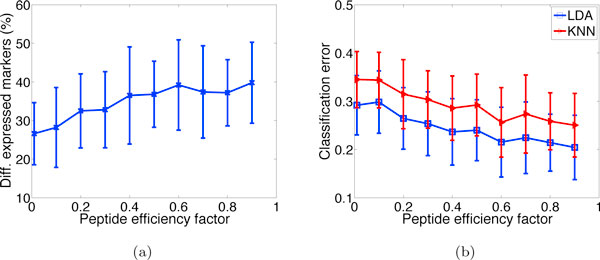
**Effect of protein abundance**. Effect of peptide efficiency factor on (a) differential expression results, and (b) classification errors for samples with reduced marker concentration. Results deteriorate compared to those using the default protein concentration (Fig. 3(b) and 3(d)).

To demonstrate the effect of protein abundance on the detection of low-abundance marker proteins, we conduct an experiment where all markers are exclusively designed to have low abundance, distributed in the lower 25% quantile of the Gamma distribution; see Eq. (1). Figure 4 depicts the corresponding plots to Figure 3(b) and 3(d) in the case of the low-abundance markers. It can be observed that the percentage of detected differentially expressed markers and the classification results become worse compared to the results in Figure 3(b) and 3(d). On average, the number of detected markers drops by 33.3% and the classification error increases by 42.4%. Similar trends are observed under other parameter settings (data not shown).

These results indicate that it is essential to develop methods to enhance the identification results of low abundance peptides which are often of more biological interests. Relative to hardware, sample fractionation and protein depletion through immunoaffinity-based approaches [31] can be helpful. Relative to software, there exist algorithms shown to be efficient for the detection of low-abundance peptides, such as BPDA2d [23].

#### Effect of sample size

**Figure 5:**
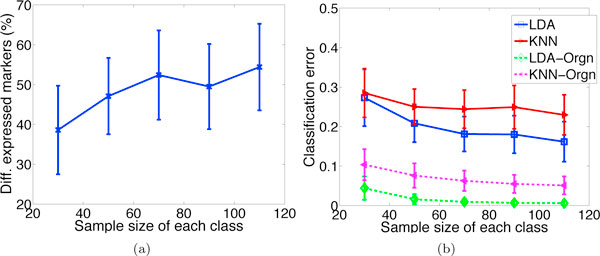
**Effect of sample size**. Effect of sample size *M *on (a) differential expression results, and (b) classification error rates. All results generally improve as *M *increases. In (b) the classification error of the original protein sample (dashed lines) is plotted side by side with that of the observed protein data (solid lines), illustrating the substantial loss in accuracy introduced by the MS analysis pipeline.

Figure 5 shows the effect of sample size. The range of values used is typical in proteomic experiments. It is observed that as more samples become available, the differential expression results and the classification accuracy improve notably. For instance, when sample size increases from 30 to 110, the number of detected markers increases by 41% and the classification error decreases by 40%.

In Figure 5(b), the classification error of the (unobserved) original protein sample, before passing through the MS pipeline, is plotted side by side with that of the observed protein data, after analysis by the MS pipeline. The performance degradation caused by various noise conditions throughout the pipeline is clearly visible.

### Instrument characteristics

#### Effect of instrument response

**Figure 6:**
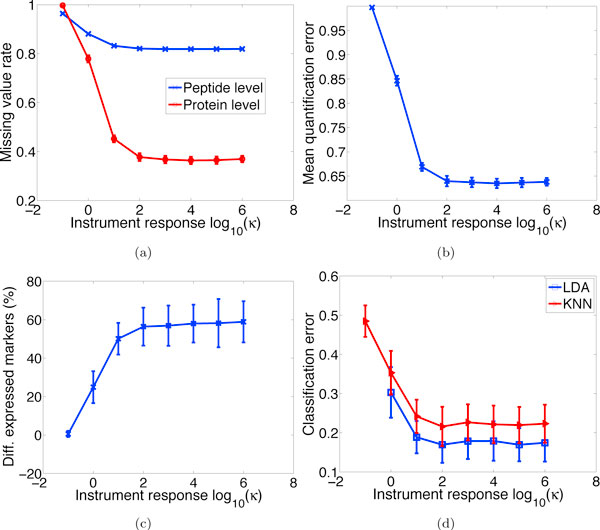
**Effect of instrument response**. Effect of instrument response factor *κ *on (a) missing value rates, (b) quantification accuracy, (c) differential expression results, and (d) classification error rates. As *κ *increases, all performance indices improve quickly and then level off.

The effect of instrument response factor *κ *is displayed in Figure 6. The experimental value of *κ *spans seven orders of magnitude. As *κ *first increases (from 0.1 to 100), true signals get amplified and SNRs become better, resulting in fewer missing values and false negatives at both peptide and protein levels (Figure 6(a)), which in turn render better quantification and differential expression results (Figures 6(b) and 6(c)). But when *κ *> 100, various performance indices level off. This illustrates that beyond a certain point, merely boosting the instrument response factor cannot help produce enhanced results. Rather, the performance bottleneck is determined by other factors such as noise in the system and efficiency of peptide detection algorithms.

#### Effect of saturation

**Figure 7:**
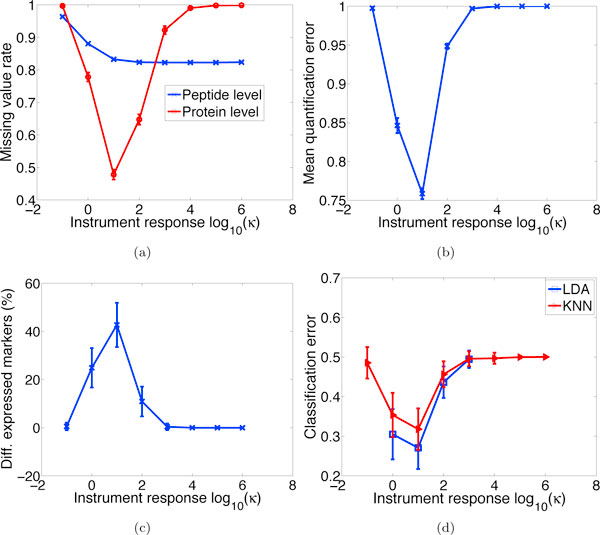
**Effect of saturation**. Effect of instrument response *κ *in the presence of saturation on (a) missing value rates, (b) quantification accuracy, (c) differential expression results, and (d) classification error rates. As *κ *increases, all performance indices at first improve and then deteriorate (except for the peptide missing value rate, which levels off).

In the previous experiment, the MS instrument is assumed to be working in the linear range. But for complex samples, for which analyte concentrations span orders of magnitude, saturation effects need to be taken into account (see Figure 2). The previous experiment is repeated with the same settings, except that the saturation upper limit *sat *is changed from infinity to 10^4^, corresponding to a 10^4 ^linear dynamic range when *κ *= 1. Interestingly, the resulting plots shown in Figure 7 are no longer monotone as observed in Figure 6. As the instrument response *κ *increases, the linear dynamic range (LDR) actually shrinks given the saturation ceiling is fixed (LDR can be approximated by *sat/κ*). Therefore, the percentage of peptides with saturated ion signals increases, and fewer peptides can pass the correlation filter, adversely affecting protein detection, quantification, and classification. To wit, when *κ >*10, the protein missing value rate shoots up, fewer markers get detected, and classification performance and protein quantification results deteriorate.

The compound effects of instrument sensitivity and saturation demonstrate that the effectiveness of MS in quantitative analysis relies on achieving a wide linear dynamic range with a high saturation ceiling and a matching sensitivity. For example, in electrospray ionization mass spectrometry, the linear range may be extended by enhancing gas-phase analyte charging, facilitating droplet evaporation, or introducing ionization competitors [32].

#### Effect of noise

**Figure 8:**
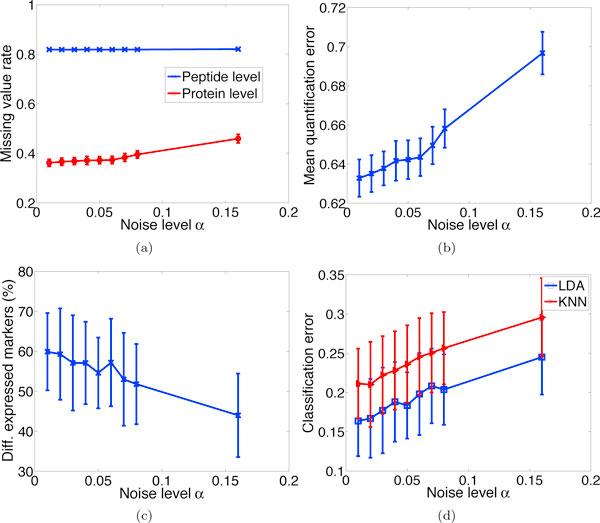
**Effect of noise**. Effect of noise on (a) missing value rates, (b) quantification accuracy, (c) differential expression results, and (d) classification error rates. The x-axis represents *α *in the noise model given by Eqs. (7)-(8), while β is set to be 120*α*. The parameter values in the middle of the range (*α *= 0.04, *β *= 4.8) were estimated by an LC-MS analysis of human serum samples [19].

Noise in the MS analysis pipeline and the performance of peptide detection algorithms affect the number of proteins that can be quantified. To study noise impact directly, we eliminate the confounding effects of the peptide detection algorithm by assuming perfect detection, with *TPR *≡ 1 for *SNR >*0 and *TPR *= 0 for *SNR *= 0. It is observed in Figure 8(a) that the peptide missing value rate stays relatively flat except at the end points where the accumulated effects of increasing noise levels are discernable: more of the true signal is obscured by noise and more peptides have infinitesimal SNR, which prevent their detection. The increasing trend in missing value rate at the protein level is more apparent: the fact that less proteins can be quantified as the noise level increases is not only due to fewer detectable peptides, but also because fewer peptides can pass the correlation filter for a protein to be quantified. Figures 8(b), (c) and 8(d) elucidate the adverse effects of noise on quantification accuracy, differential expression and classification results, respectively.

### Peptide detection and experimental design characteristics

#### Effect of MS1 peptide detection algorithm

**Figure 9:**
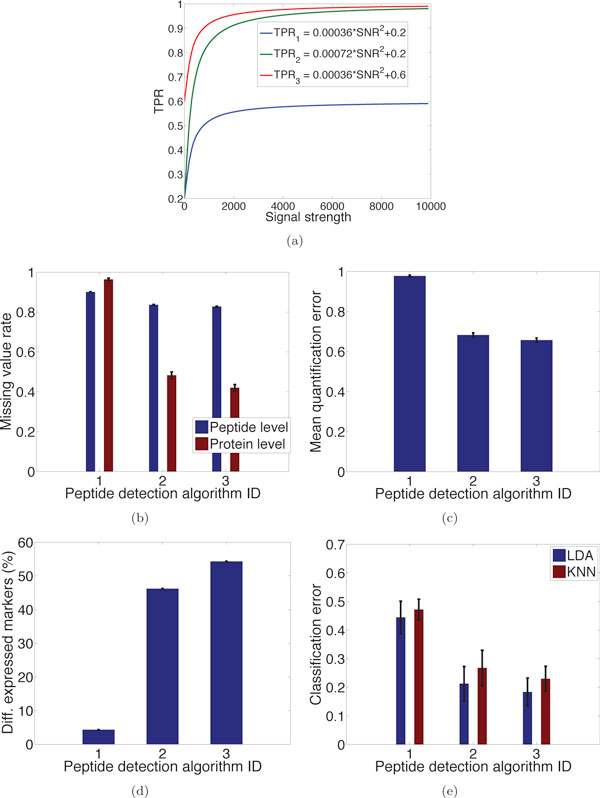
**Effect of peptide detection algorithm**. Effect of using three hypothetic detection algorithms with increasingly better performance, quantified by the (a) TPR vs. signal strength curves. The applications of the three algorithms lead to increasingly improved results in terms of (b) missing value rates, (c) quantification accuracy, (d) percentage of detectable markers, and (e) classification error rates.

Given the same experimental settings, the performance of peptide detection algorithms may significantly affect the number of detected true positives (TPs). Three hypothetic detection algorithms with increasingly better performance are considered, in terms of TPR vs. signal strength curves; see Figure 9(a). It can be seen in Figure 9(b-e)) that the application of these detection algorithms leads to increasingly better results in terms of missing value rate, quantification accuracy, detectable markers, and classification performance.

#### Effect of overlapping peptides and mass resolving power

**Figure 10:**
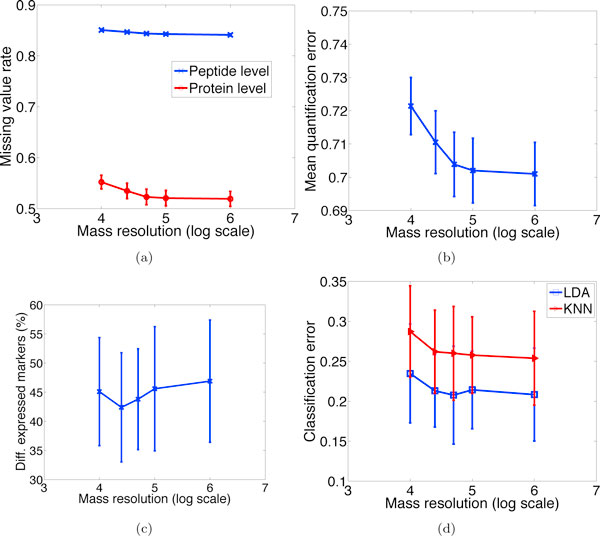
**Effect of mass resolving power**. Performance of a typical peptide detection algorithm in the second category described in the text under various mass resolutions and in the presence of overlapping peptides. (a) Missing value rates, (b) quantification accuracy, (c) differential expression results, and (d) classification errors.

To quantitatively evaluate the performance of MS1-based peptide detection algorithms under various mass resolutions and in the presence of overlapping peptides, two categories of detection algorithms are compared: the first characterizes those which can effectively detect convoluted peptides, such as NITPICK [24], BPDA [22] and BPDA2d [23], which are modeled by an overlapping factor *o_ij _*= 1 in Eq. (10), and the second represents those that are sensitive to mass resolution and ineffective in detecting overlapping peptides (e.g. algorithms based on greedy template-matching), which are modeled by letting *o_ij _*be inversely proportional to the number of overlapping peptides with peptide *i *in sample *j*. For algorithms in the first category, robust performance is expected for a range of mass resolutions (data not shown). In contrast, for algorithms in the second category, various performance indices generally become worse as mass resolving power declines, since more peptides cannot be resolved and are lost in detection (see Figure 10). Summing up, the superiority of the first category over the second will be more evident for complex samples with more proteins and co-eluting analytes analyzed by a MS instrument with limited mass resolution.

#### Effect of MS2 replication

In tandem MS analysis, the precursor ions selected for fragmentation have low reproducibility across runs, and only a subset of peptides present in the sample can be analyzed for each run; this problem is known variously as MS2 random sampling and MS2 under-sampling [33]. Hence, though laborious and costly, replicate MS2 measurements are frequently conducted for in-depth proteomic profiling or for building an AMT database to facilitate quantitative and high-throughput proteome measurements [34].

**Figure 11:**
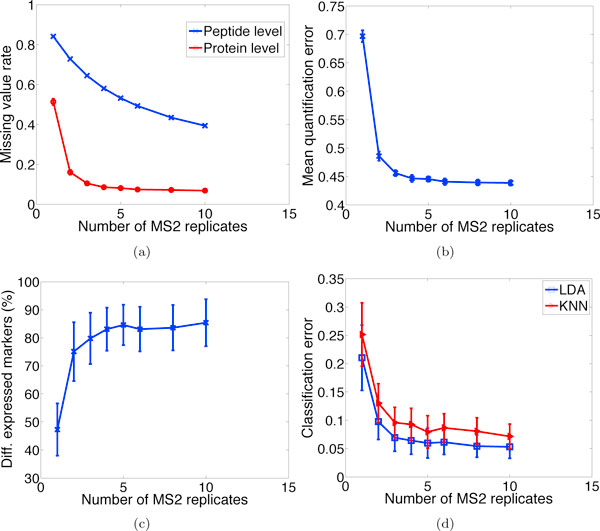
**Effect of MS2 replication**. Effect of MS2 replication on (a) missing value rates, (b) quantification accuracy, (c) differential expression results, and (d) classification errors. It can be seen that replicate analysis can significantly boost peptide and protein identification rates, quantification and classification results even only a few replicates are made available.

The effect of MS2 replication on various performance metrics is illustrated in Figure 11. It is observed that even with a few replicate assays (as low as two or three), peptide and protein identification rates are remarkably boosted. As more replicates are made available, the protein identification rate levels off faster than the peptide rate, which was also observed in [29], indicating that newly identified peptides are mostly associated with already identified proteins. This may be explained as a bias towards relatively easily detectable proteins. Those proteins that are hard to detect may be a result of degradation, a spare amount of children peptides, ineffective ionization, and so on. Figures 11(a) and 11(b) show that more proteins are detectable with improved quantification accuracy as the number of replicates increase. Comparing the use of three replicates against a single assay, Figure 11(c) shows that the number of detected differentially-expressed marker proteins nearly doubles, while Figure 11(d) indicates that the LDA classification error enjoys a 67% decrease.

### Summary

The median value of each performance index across all previously studied cases with default sample size 100 is given in Table 2. It can be seen that the protein quantification rate exceeds the peptide identification rate. This may be explained by the one-to-many map from protein to its digested peptides: a protein can be quantified if more than one of its children peptides are identified and can pass the aforementioned quality filter. In the proteome studied, on average, one protein can be digested into around 20 peptides, and if we simply assume that each child peptide can be identified with a probability 0.17 (the calculated average peptide identification rate), independent of other peptides, and ignore the additional effects of the quality filter, then the protein quantification probability (an upper bound) can be approximated by 1 - (1 - 0.17)^20 ^- 20 × 0.17 × (1 - 0.17)^19 ^= 0.88. The typical percentage of detected differentially-expressed protein markers is around 50% and the median value of the LDA classification error on the observed protein data is 0.18, which is 17 times larger than that of the original protein data -- this exemplifies the signal corruption and error propagation introduced by the MS analysis pipeline, as well as the intricacy of biomarker discovery and their applications in disease diagnosis due to limited sample size, signal interference, ubiquitous noise, measurement errors, and so on.

**Table 2:** Results summary

Performance indices	Median values
Peptide identification rate	0.17
Protein quantification rate	0.54
Protein quantification error	0.67
Percentage of detected markers	52%
LDA error on the original protein data	0.01
KNN error on the original protein data	0.03
LDA error on the observed protein data	0.18
KNN error on the observed protein data	0.24

## Conclusion

We have identified and analyzed different modules in a typical MS based proteomic work flow, resulting in a proteomic pipeline model that captures key factors in system performance. Through simulation based on ground-truthed synthetic data, we studied the effect of the various model parameters on the number of identified peptides and quantified proteins, quantification errors, detectable differentially expressed protein markers, and classification performance.

The main observations that were gleaned from the results of this study are as follows.

• Regarding sample characteristics, we observed a positive correlation between peptide efficiency and performance. The intricacy in detecting low-abundance peptides was demonstrated, thereby elucidating the advantage of sample fractionation and protein depletion through immunoaffinity-based approaches. Moreover, we showed that results could be improved by increasing sample size.

• As for instrument characteristics, the compound effects of instrument response and saturation were first examined and it was shown that the effectiveness of MS in quantitative analysis relies on achieving a wide linear dynamic range with a high saturation ceiling and matching instrument sensitivity. Enhancing gas-phase analyte charging, facilitating droplet evaporation, or introducing ionization competitors can be beneficial in extending the linear dynamic range. The adverse effects of noise was illustrated, highlighting the need in strictly following experiment protocols to minimize variance and measurement error.

• Peptide detection and experimental design characteristics were also studied. It was shown that improving peptide detection algorithms in the direction of enhancing true positive rate for a wide range of SNR (especially for low SNR) and tackling convoluted peptide signals could be invaluable, especially for complex samples and for MS instruments with limited mass resolution. It was also observed that the use of only a small number of replicate tandem MS assays could effectively reduce the MS2 under-sampling problem and improve performance.

To enable the performance analysis of such a complex system, many reasonable assumptions are made and the pipeline is simplified and reduced to a few key characteristics; nevertheless corruption of the true signal caused by the pipeline is evident and readily seen. This is expected to become worse as more steps are considered.

Though we used two sample types to illustrate the use of the LC-MS based pipeline model, the extension to multiple sample types is straightforward. In addition, the same methodology can be applied to study other MS platforms such as matrix-assisted laser desorption/ionization (MALDI). In addition, a similar strategy applies to labeled experiments.

The proposed pipeline model can be used to optimize the work flow and to pinpoint critical steps to which it is worth allocating resources in order to improve biomarker detection performance, thereby giving it wide application potential in the current drive to enable proteomic biomarker discovery from MS data.

## Competing interests

The authors declare that they have no competing interests.

## Authors' contributions

YS developed and implemented the pipeline model, conducted all simulations, and wrote the initial draft of the paper. UBN proposed the use of the pipeline model, advised YS on the numerical experiments, and revised the paper. ERD revised the paper. All authors read and approved the final manuscript.
